# Improving Exposure Assessment Methodologies for Epidemiological Studies on Pesticides: Study Protocol

**DOI:** 10.2196/16448

**Published:** 2020-02-28

**Authors:** Kate Jones, Ioannis Basinas, Hans Kromhout, Martie van Tongeren, Anne-Helen Harding, John W Cherrie, Andrew Povey, Zulkhairul Naim Sidek Ahmad, Samuel Fuhrimann, Johan Ohlander, Roel Vermeulen, Karen S Galea

**Affiliations:** 1 Health and Safety Executive Buxton United Kingdom; 2 Centre for Human Exposure Science Institute of Occupational Medicine Edinburgh United Kingdom; 3 Institute for Risk Assessment Sciences Utrecht University Utrecht Netherlands; 4 Centre for Occupational and Environmental Health School of Health Sciences, Faculty of Biology, Medicine and Health University of Manchester Manchester United Kingdom; 5 Institute of Biological Chemistry, Biophysics and Bioengineering Heriot Watt University Edinburgh United Kingdom

**Keywords:** pesticides, occupational exposure, epidemiology, algorithm, biomonitoring, urine, questionnaire

## Abstract

**Background:**

Exposure to certain pesticides has been associated with several chronic diseases. However, to determine the role of pesticides in the causation of such diseases, an assessment of historical exposures is required. Exposure measurement data are rarely available; therefore, assessment of historical exposures is frequently based on surrogate self-reported information, which has inherent limitations. Understanding the performance of the applied surrogate measures in the exposure assessment of pesticides is therefore important to allow proper evaluation of the risks.

**Objective:**

The *Improving Exposure Assessment Methodologies for Epidemiological Studies on Pesticides* (IMPRESS) project aims to assess the reliability and external validity of the surrogate measures used to assign exposure within individuals or groups of individuals, which are frequently based on self-reported data on exposure determinants. IMPRESS will also evaluate the size of recall bias on the misclassification of exposure to pesticides; this in turn will affect epidemiological estimates of the effect of pesticides on human health.

**Methods:**

The IMPRESS project will recruit existing cohort participants from previous and ongoing research studies primarily of epidemiological origin from Malaysia, Uganda, and the United Kingdom. Consenting participants of each cohort will be reinterviewed using an amended version of the original questionnaire addressing pesticide use characteristics administered to that cohort. The format and relevant questions will be retained but some extraneous questions from the original (eg, relating to health) will be excluded for ethical and practical reasons. The reliability of pesticide exposure recall over different time periods (<2 years, 6-12 years, and >15 years) will then be evaluated. Where the original cohort study is still ongoing, participants will also be asked if they wish to take part in a new exposure biomonitoring survey, which involves them providing urine samples for pesticide metabolite analysis and completing questionnaire information regarding their work activities at the time of sampling. The participant’s level of exposure to pesticides will be determined by analyzing the collected urine samples for selected pesticide metabolites. The biomonitoring measurement results will be used to assess the performance of algorithm-based exposure assessment methods used in epidemiological studies to estimate individual exposures during application and re-entry work.

**Results:**

The project was funded in September 2017. Enrollment and sample collection was completed for Malaysia in 2019 and is on-going for Uganda and the United Kingdom. Sample and data analysis will proceed in 2020 and the first results are expected to be submitted for publication in 2021.

**Conclusions:**

The study will evaluate the consistency of questionnaire data and accuracy of current algorithms in assessing pesticide exposures. It will indicate where amendments can be made to better capture exposure data for future epidemiology studies and thus improve the reliability of exposure-disease associations.

**International Registered Report Identifier (IRRID):**

PRR1-10.2196/16448

## Introduction

### Background

Exposure to certain pesticides has been implicated in the development of several chronic diseases such as some cancers, respiratory effects, reproductive effects, and Parkinsonism [1-5]. Determining any role of pesticides in chronic health diseases requires the assessment of historical exposures. However, exposure measurement data are rarely available to adequately cover the entire exposure time period. Therefore, assessment of historical exposures is frequently based on self-reported surrogate information such as a person’s job title, duration of employment, whether they were ever exposed (yes or no) to pesticides. Naturally, such exposure measures have limitations, for example, the ability of a person to remember all their jobs, which may affect the conclusions of a study [6]. The large number of pesticide active ingredients and pesticide mixtures involved, their different toxicokinetics, seasonality of use, and a broad range of characteristics regarding their application and use further complicate the assessment of workers’ exposure to pesticides as it is difficult to accommodate all these factors in a modeled assessment. Pesticide exposure intensity has also been understudied or underaccounted for but may be an important factor [7]; much of the current literature focusses on cumulative lifetime exposure. Understanding the performance of the applied surrogate measures in exposure assessment is therefore important to allow proper estimation of the risks.

*Improving Exposure Assessment Methodologies for Epidemiological Studies on Pesticides* (IMPRESS) is a collaborative project between the Institute of Occupational Medicine (IOM), the Health and Safety Executive’s (HSE’s) laboratory, the Institute of Risk Assessment Sciences at Utrecht University, and the Centre for Occupational and Environmental Health of the University of Manchester. The overall study seeks to improve understanding of the performance of pesticide exposure assessment methods (EAMs) used in previous epidemiological investigations, and to use this information to recommend enhancements in scientific practice for future studies. For this, the project will assess the reliability and external validity of the surrogate measures used to assign exposure within individuals or groups of individuals. Moreover, the size and impact of recall bias on the misclassification of exposure to pesticides and the associated health effects will be evaluated. Previous and newly collected exposure data from several existing epidemiological studies across 3 continents (including quantitative exposure measurements using biological monitoring methods) will be used in these evaluations. IMPRESS will also assess the performance of various EAMs used in epidemiology by comparing and contrasting them within existing epidemiological studies. A dedicated systematic review was performed to assist in the selection of relevant methods to be included in these comparisons [8].

The main outcomes of the IMPRESS project will include the following:

1. Mapping of the methods used for exposure assessment in occupational epidemiological studies [8];
2. An assessment of the ability of workers to remember their working history related to pesticide exposure over a range of time frames;
3. Evaluation of an easily adaptable semiquantitative individual-based EAM against measured levels of urine pesticide metabolites in a broad range of settings and;
4. The comparison of the reliability and performance of EAMs used to assign exposure to individuals (individual-based) or groups of individuals sharing common attributes (group-based) in the frame of existing epidemiological studies and against the same exposure history and health outcome data.

### Protocol Aims and Objectives

This protocol outlines the methods to achieve the following 2 project aims:

1. Evaluate recall of exposure to pesticides and information on exposure determinants to estimate the size of any recall bias and its effect on misclassification in a few specific pesticide-using populations (described in Table 1). The primary mechanism for this will be to reinterview workers already enrolled within the existing epidemiological cohorts. This addresses outcome 2 above. As already mentioned, many epidemiological studies rely on questionnaire data to determine exposure and so the reliability of such recall is crucial to understanding the validity of the conclusions reported in such studies. This is referred to as recall bias subsequently in this paper.
2. Examine the reliability and validity of currently available individual-based EAMs for pesticide exposure. The main approach for this will be the collection and analysis of urine samples for selected pesticide metabolites from participants alongside details about the pesticide use. The derived results will be used as a comparative measure for the evaluation of the performance of the individual-based EAMs. This addresses outcome 3 and provides with a reference method for the benchmarking exercise included in outcome 4 above. The best studied algorithm (the Agricultural Health Study, AHS) was developed for US-style farming exposures; it is not clear how suitable this algorithm is for other farming systems, such as small-scale (the United Kingdom) and low and middle income countries. IMPRESS will assess both of these situations. This is referred to as exposure assessment subsequently in this paper.

This protocol will be applied in a number of epidemiological studies, which are detailed below.

Briefly, the first project aim (point 1: recall bias) will be applied in the UK (using an ongoing epidemiological cohort (Prospective Investigation of Pesticide Applicators’ Health [PIPAH] [9,10]), 2 historical cohorts (Pesticide Users Health Study [PUHS] [11] and Study of Health in Agricultural Work [SHAW] [7]) that analyzed the association between low-dose pesticide exposure and neuropsychiatric disorders [12] and some historical biomonitoring data [13]), and a study among Ugandan farmers (Pesticide use in tropical settings [PESTROP]) examining the association between pesticide exposure and health including the identification of methods for exposure prevention [14]. The second project aim (point 2: exposure assessment) will be applied in the UK PIPAH cohort, the Ugandan PESTROP cohort and a Malaysian cohort of farmers (Ahmad, personal communication).

**Table 1:** Existing studies to be included in *Improving Exposure Assessment Methodologies for Epidemiological Studies on Pesticides*.

Study	Project aims	Potential participants, N	Date of OQ^a^
UK Prospective Investigation of Pesticide Applicators’ Health	Recall bias and exposure assessment	825 certified pesticide users	OQ 2016
UK Pesticide Users Health Study	Recall bias and exposure assessment	>500 certified pesticide users	OQ 2004-2006
UK Study of Health in Agricultural Work	Recall bias only	Up to 234 farmers	OQ 2002
UK Historical biomonitoring data	Recall bias only	Up to 115 pest control operatives, tree dippers, and orchard sprayers	No OQ, standardized one to be used
Malaysia	Exposure assessment only	150 small-scale farmers	No OQ
Uganda	Recall bias and exposure assessment	300 small-scale farmers	OQ 2017

^a^OQ: original questionnaire.

### Prospective Investigation of Pesticide Applicators' Health Study

The UK PIPAH Study was established in 2013, with the aim of investigating whether there is any evidence of a link between working with pesticides and health. Men and women who are certified pesticide users are eligible to join the study. All the members of the National Register of Sprayer Operators (NRoSO) and the National Amenity Sprayer Operators’ Register were invited to take part in the study. Members of HSE’s other long-term health study on pesticides, the PUHS, were invited to join in 2014. Over 5700 baseline questionnaires have been completed to date and enrollment is ongoing. A pesticide use questionnaire was sent to the whole cohort in January 2017 with about 1500 responses received. We propose to recruit from the 825 NRoSO respondents to that questionnaire (asking about their pesticide use in 2016), excluding any PUHS recruits, who will instead be invited to participate in a rerun of the 2004-06 questionnaire (see below).

### Pesticide Users' Health Study

The PUHS was established by HSE in the late 1990s. The aims of the study are to monitor the long-term health of individuals potentially exposed to low levels of pesticides on a long-term basis. From 1994 to 2003, anyone applying for certification (required by users of agricultural pesticides under the Control of Pesticides Regulations 1986) was invited to give their permission for HSE to access information relating to them for the purpose of medical research into pesticide use. Those who agreed became members of the PUHS (around 65,000 participants). From 2004 to 2006, HSE sent a questionnaire to all participants. As this is a historical cohort, only those participants who have been subsequently recruited into the PIPAH study and are currently active pesticide users will be contacted (>500 participants).

### Study of Health in Agricultural Work

The SHAW was a study that commenced in 2002, designed to address the question of whether low-dose pesticide exposure was associated with neuropsychiatric disorders in UK farmers. A cohort of British farmers working in the 1970s was sent a screening questionnaire which asked about their health and work history. Questionnaires were returned from 1380 subjects; there was evidence that handling the pesticide concentrate for the treatment of sheep was associated with screen-positive ill health [7]. A subgroup of this cohort (n=234) was interviewed to obtain more detailed information on ill health and exposure history. This smaller group will form the basis of the recall bias recruitment. It should be noted that a substantial proportion of this population may have died since the original study, and hence pilot work will establish to what extent this population is still alive and are willing to participate.

### United Kingdom Historical Biomonitoring Data

HSE has conducted a number of research projects looking at pesticide exposures in a range of sectors, using biological monitoring as an estimate of exposure. Suitable historical studies were identified: a permethrin survey (1992-93) looking at pest control operatives (N=30) and tree dippers (N=22), and an orchard spraying survey (1996-97; N=63). Although the original numbers (and therefore the number of likely respondents some 20 years later) are small in each case, the use of a standardized questionnaire should allow some use of pooled responses and comparison with the same questions that appear in the newer cohorts.

### The Malaysian Farmers Study

The Malaysian farmer’s study is a prospective study of farmer’s ill health in the pesticide spraying season in the Sabah region of Malaysia, which started in 2018. Farmers (approximately 150) were randomly selected from regional databases of farmers and were interviewed to provide baseline information on sociodemographic and occupational factors as well as their health. During the spraying season, farmers will collect spot urine samples, be observed (with videoing) by a trained researcher, and keep a diary on pesticide use and health symptoms.

### Uganda: Pesticide Use in Tropical Settings

The PESTROP study consists of 300 smallholder farmers who were interviewed twice within an interval of 2 to 4 weeks in 2017 [14]. A structured questionnaire was used to obtain insights on sociodemographics, knowledge, attitude, and practices of pesticide use and corresponding protective behavior, as well as health history. An adapted pesticide exposure algorithm was developed [15]. In addition, a neurobehavioral test battery (eg, Purdue Pegboard and Finger Tapping Test) was administered, anthropometry (height, weight, and waist circumference) was recorded, erythrocytic acetylcholinesterase activity was measured, and urine, hair, and toenail samples were collected.

The protocol will be adapted as necessary to accommodate the specific requirements of each of these cohorts, with separate ethics approvals being sought.

## Methods

### Study Design

#### Overview

**Figure 1:**
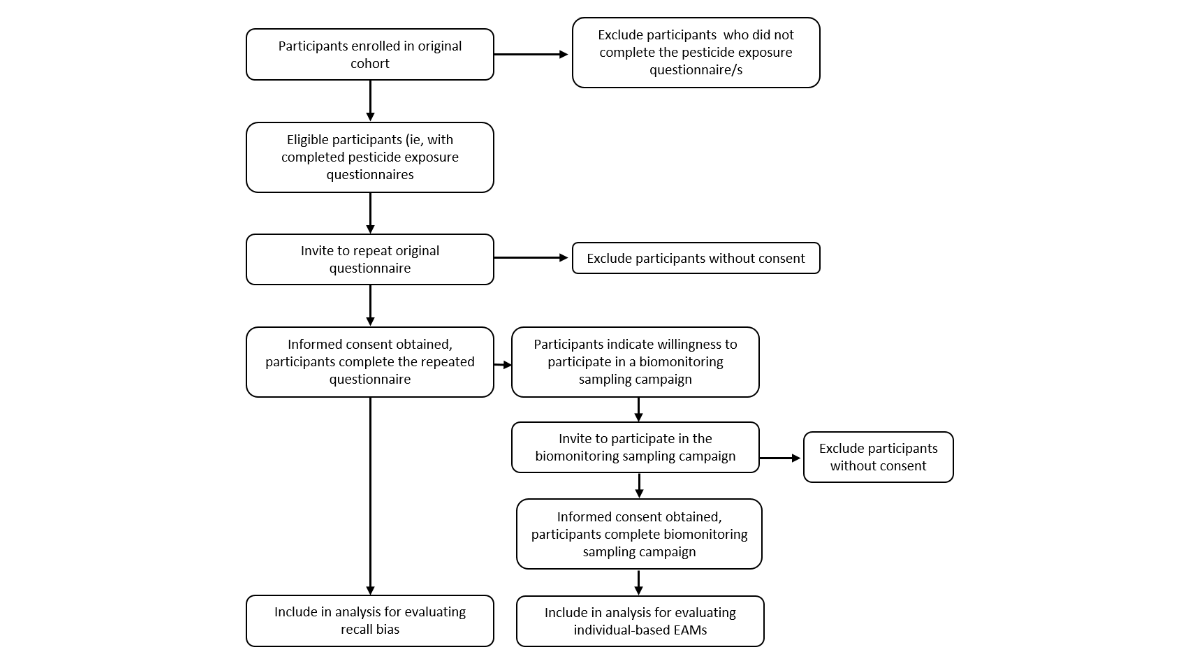
Summary of enrollment and data collection process. EAM: exposure assessment method.

For the epidemiological studies involved in both project aims (points 1 and 2 above, ie, PIPAH and Ugandan studies), participants will be recruited to address the first project aim (recall bias) and then invited to participate in the second project aim (exposure assessment). Figure 1 provides a schematic description of the enrollment and data collection process.

#### Identification and Recruitment of Participants

Cohort participants who are aged 18 years or over, who are (*were* for SHAW and those included in the historical biomonitoring data cohorts) occupationally active in a job that involved direct (ie, handling or application) or indirect (ie, re-entry) exposure to pesticides during the original study period and completed the pesticide use questionnaire in the original study will be contacted to agree to complete a questionnaire relating to their previous participation in the cohort (all except the Malaysian cohort and the historical biomonitoring data), allow any data sources relating to them in the other identified cohorts to be combined (PUHS and PIPAH participants only) and, to be contacted concerning participation in the new cohort sampling as part of the second (exposure assessment) project aim (PIPAH study only, Malaysian and Ugandan participants recruited independently).

### United Kingdom Cohorts

For the UK cohorts (PUHS, PIPAH, SHAW, and historical biomonitoring study data), survey packs will be sent out in mid-2019 to individuals fulfilling the inclusion criteria. Each survey pack will be customized to the particular cohort and will contain a letter of invitation, a participant information sheet, a consent form, a postage paid return envelope, and (for the PUHS, PIPAH, and historical biomonitoring data groups) the questionnaire. The SHAW questionnaire is to be conducted by telephone interview and so will not be included in the survey pack, although the participants will be sent a copy of their work history from the time (mirroring the previous study).

Potential SHAW participants will be invited to provide their written informed consent to participate in the study, returning this in the postage-paid return envelope. For those who do not respond to the first mailing, a reminder survey pack will be sent to them within 3 months. For better consistency with the initial administration method (in-person interview), participants will be interviewed by telephone based on the original questionnaire survey material (it is not practical to reinterview face to face). It is anticipated that the telephone interview will take up to 1 hour to complete.

Potential PUHS, PIPAH, and historical biomonitoring study data participants will be invited to provide their written informed consent to participate in the study and complete the paper questionnaire sent to them, returning both of these in the postage-paid return envelope. For those who do not respond to the first mailing, a reminder survey pack will be sent within 3 months. Participants who give consent to take part in the second (exposure assessment) project aim will then be sent a new survey pack containing a letter of invitation, a participant information sheet, a consent form, a sampling kit with activity diary, and a postage paid return envelope.

### Ugandan Cohort

For the Ugandan cohort, a local researcher will administer the survey material to participants who provided written informed consent, as per the original cohort study design (Fuhrimann, personal communication). The completion of the questionnaire will take place at the farm or workplace where participants are recruited. Later, participants will be invited to take part in the exposure assessment study, which will be conducted on a day when participants report spraying pesticides.

### Malaysian Cohort

For the Malaysian cohort, a local researcher will supervise the urine sample collection and activity diary completion, as per the cohort study design. The completion will take place at the workplace where participants are recruited.

### Data Collection

#### Evaluation of Recall Bias

Consented participants will be requestioned concerning the exposure information they had previously provided as part of the pesticide use questionnaires administered. Requestioning participants about both relatively recent and historical exposures will enable assessment of the consistency of recall where common questions exist across studies. Time frames for recall are 2 years (PIPAH and the Ugandan cohort), 6-12 years (PUHS), and up to 28 years (SHAW and historical biomonitoring study data). Participants will be administered a similar questionnaire as used previously (with the exception of those in the historical biomonitoring study), with slight modifications to highlight the time periods of interest to the IMPRESS project. In addition, all farmers in SHAW, in line with the original study protocol, will be administered the memory section of the Cambridge Cognition Examination instrument to allow for an assessment of their memory function [16]. The format and relevant questions will be retained but some extraneous questions from the original questionnaire (eg, relating to health) will be excluded for ethical (unused information) and practical (time taken to complete) reasons. The questionnaires will be completed in the same manner (or as near to) as previously administered to avoid any potential bias in their completion owing to different methodology being used. All written questionnaires are expected to take around 20 min to complete.

#### Evaluation of Currently Available Individual-Based Exposure Assessment Methods for Pesticide Exposure

Urine samples will be collected during the spraying season. Participants will be asked to provide samples after being involved in the use of, or indirect contact with, one or more pesticides. Activities targeted will be handling, spraying and re-entry with each participant providing samples for 1 of the 2 tasks. In general, sampling will occur irrespective of the pesticide involved except for the UK participants. UK participants will be asked to collect samples, if possible, when a pesticide from a list of preselected substances is used (see Table 1 for details). However, if those products are not routinely used, then UK participants will be asked to provide samples on any day when there is contact with pesticides and to record the product or active ingredients in the activity diary. This approach is intended to preserve statistical power by minimizing the number of different substances or metabolites measured while accounting for logistical constraints (ie, time spent in the field for studies where physical presence of investigators is required) and resources required for sampling material, storage, and analysis.

For every activity of interest, a spot sampling strategy of a pre- and a postactivity urine sample from each consenting participant will be followed. Preactivity samples will be collected before the activity commences (usually early in the morning), whereas postactivity samples will generally be evening voids. In the United Kingdom, postactivity samples will be collected at a standard time frame defined as between 6.00 pm and 8.00 pm. For re-entry tasks, sample collection will be attempted within 7 days of the performance of a crop spraying activity. Clear instructions on how to provide the urine samples in a manner to minimize potential cross-contamination will be given in a written (UK) or verbal (Uganda and Malaysia) and semipictorial form (all). Field blanks will be collected to assess any contamination of sample bottles by the worker. These will comprise empty vials, filled with tap or bottled water by the participants themselves, and will be included in approximately 10% of the samplings, with selection being made at random by the researcher.

Researcher-led (Uganda and Malaysia) or self-administered (the United Kingdom) diaries will be used to collect information on factors identified in the literature as important for determining the workers’ level of pesticide exposure. This will include contextual information, for example, on activities involved and time spent on them, pesticide application and mixing methods, equipment used, where activities take place (indoor or outdoor), cleaning, products and quantities used, and use of personal protective equipment (PPE). In the Malaysian study, farmers are also to be videorecorded during their normal working practices.

Each study pack provided to UK participants will include urine sample receptacles and appropriate packaging, a leaflet with simple instructions for the collection of the urine samples, the related activity diary, and a prepaid envelope to return the material. Study packs for non-UK participants will be in line with the developed in-study protocols including the urine sample receptacles, collection bags, and relevant guidelines whenever applicable.

### Urine Sample Handling and Analysis

Laboratory analysis for all cohorts (including physical preparation, storage, and out of field handling and processing of the urine samples and collection materials) will be performed at the facilities of HSE’s laboratory by dedicated and well-trained personnel of the institution.

#### Labeling and Tracking Samples

A comprehensive labeling and tracking system will be implemented to ensure that the contextual information from the collected diaries and questionnaires is clearly linked to the urine sample results and the participant. Given that the study population is sourced from ongoing epidemiological studies, existing identity numbers assigned to the participants are expected to play a key role on this sample tracking system.

Each study pack and included material (ie, diary, return envelope, and urine receptacles) will be prelabeled with a unique identifying number before being issued. This will include the (existing) participant’s study ID, along with a sample number to reflect each consecutive sample provided with a prefix indicating the country population concerned (ie, UK [United Kingdom], UG [Uganda], and MY [Malaysia])—for example, UKXXXX-01 is the preactivity sample for UK participant ID number XXXX; UKXXXX-02 is the postactivity sample for the same participant.

#### Urine Sample Storage and Transportation

For UK participants, administration of urine samples and related survey material will be made by first class post as per standard practice, with prestamped envelopes for returning the materials being provided as part of the survey pack.

For Ugandan and Malaysian participants, urine sample receptacles will be provided at the time of the questionnaire interviews conducted according to the specific in-study designs. For Uganda, this will be in conjunction with the interviews performed as part of the first project aim (recall bias). Retrieval of the collected samples for these workers will be performed by the investigators. The researcher will log details of the samples, and the samples will be stored in a freezer at temperatures less than −15°C until transfer to the HSE's Laboratory in the UK for analysis. Transfer of samples will be performed in batches at intervals regulated by number of collected samples. The samples will be provided to the courier service responsible for the transfer in frozen study packs inside cool boxes with dry ice, thereby maintaining the cold chain (confirmed by an included data logger).

During every stage of the process, care will be taken to ensure that handling and transportation of the collected samples is undertaken in accordance with well-established protocols specific for the involved studies; this includes field blanks and spikes and stability testing [17]. At each stage in the chain, the integrity of the labels will be checked. If, at any stage, the label on the urine sample receptacle becomes damaged, a new label with the same sample ID number will be added. For each pack, the sample numbers on the urine sample receptacle and diary will again be checked to ensure they match. Details of the urine sample will then be logged as per HSE’s laboratory standard practices, and the sample will be stored in a freezer with a temperature less than −15°C until extraction and analysis. During the storage period, conditions will be monitored with temperature entries being logged regularly.

#### Urine Sample Analysis

The collected urine samples will be analyzed for pesticide metabolite content using gas or liquid chromatography with mass spectrometric detection. The laboratory will follow ISO9001 record keeping and other relevant quality procedures. Metabolite concentrations will be expressed either as µg/L or corrected for creatinine concentration. Relevant urinary biomarkers will be selected based on the extent of use within the study populations, validity of biomonitoring methods (availability, specificity, robustness, and quality assurance), and knowledge of toxicokinetic parameters [18,19]. Analysis will follow recommendations from a recent study on this topic [20]. Preliminary information from within the participating studies (PIPAH, Uganda, and Malaysia) indicates that the pesticides listed in Table 2 are likely to be frequently used; all have well-established methods with the ability to detect low-level exposures found within general populations, so occupational exposures are expected to be readily detected. Where possible, samples will be collected according to use of the pesticides listed in Table 2. Where none of these are used, samples will be collected after pesticide use (active ingredient recorded) with a view to appropriate analysis where possible.

Quality control will be provided in the form of field blanks and spikes and laboratory spike samples prepared under standard procedures for the purpose. The field blanks and spikes will receive the same treatment as the normal samples in terms of handling, storage conditions, and analysis. Laboratory spikes serve as internal quality control material (pooled blank urine spiked with known quantities of relevant pesticide metabolite). This material will then be analyzed with every set of real samples to ensure consistency of analysis. It will also be used to determine the stability of urine samples under various conditions to represent the field situations. Where external quality assurance is available (eg, 3,5, 6-Trichloropyridinol, cis and trans isomers of 3-(2,2-dichloroethenyl)-2, 2-dimethylcyclopropane carboxylic acid, and 3-(2, 2-dibromovinyl)-2, 2-dimethyl-(1-cyclopropane) carboxylic acid; German External Quality Assessment Scheme For Analyses in Biological Materials), the laboratory will participate. Analysts will be blinded to sample status (preexposure, postexposure, field blanks, and field spikes).

All samples and results will be logged into HSE’s Biological Monitoring Database [13]. Samples will be identified by the anonymized sample identification number. The results of the urine sample analysis will be reported by sample ID number to the project team for data analysis.

**Table 2:** List of active ingredients to be prioritized for exposure assessment by biological monitoring.

Pesticide	Biomarker	Specificity
Chlorpyrifos	TCPyr^a^	Semispecific^b^
Chlorpyrifos-methyl	TCPyr	Semispecific^b^
Cypermethrin	DCVA^c^	Semispecific^d^
Deltamethrin	3-(2,2-dibromovinyl)-2,2-dimethyl-(1-cyclopropane)carboxylic acid	Specific
Glyphosate	Glyphosate	Specific
Pirimicarb	5,6-dimethyl-2-(methylamino)pyrimidin-4-ol	Specific
Lambda-Cyhalothrin	CFVA^e^	Semispecific^f^
Bifenthrin	CFVA	Semispecific^f^
Mancozeb, Maneb, and others	Ethylenethiourea	Generic
Chlormequat chloride	Chlormequat	Specific
Fluroxypyr	Fluroxypyr	Specific
Cyfluthrin	DCVA	Semispecific^d^
2-Methyl-4-chlorophenoxyacetic acid	2-Methyl-4-chlorophenoxyacetic acid	Specific
Acetamiprid	n-Desmethyl Acetamiprid	Specific

^a^TCPyr: 3,5,6-Trichloropyridinol.

^b^Specific to chlorpyrifos and chlorpyrifos-methyl.

^c^DCVA: cis and trans isomers of 3-(2,2-dichloroethenyl)-2,2-dimethylcyclopropanecarboxylic acid.

^d^Specific to permethrin, cyfluthrin, and cypermethrin (and isomers).

^e^CFVA: cis-3-(2-chloro-3,3,3-trifluoroprop-1-en-1-yl)-2,2-dimethylcyclopropane carboxylic acid.

^f^Specific to lambda-cyhalothrin and bifenthrin.

### Data Management

All data storage and handling within the project will be performed according to the specification and requirements of the European Union’s General Data Protection Regulation (GDPR) 2018. Entry of the collected questionnaire data will be manual using an interface provided as part of the original study protocol (eg, Snap Surveys Ltd for the PIPAH and PUHS cohorts) or directly into a spreadsheet (for the other groups). There will be data entry checking (10% of the total number of records in a randomly selected manner) by another researcher not previously involved in the process. If errors are found in more than 5% of the examined records (ie, approximately 1% of the total sample), then all records will be rechecked against the original hard copies. The relevant comparison data from the original questionnaires will also be added to the spreadsheet or exported into a compatible format. Once completed, data for each cohort will be anonymized and exported into MS Excel or coma-separated value database before being transferred to IOM (in accordance with a project Data Transfer Agreement and the GDPR).

Access to identifiable information about an individual will be restricted to the institution responsible for the particular cohort and available only to a limited number of authorized employees responsible for administering their cohort. Hard copies of questionnaires and survey material will be securely stored by that institution. Any electronic files of questionnaires and other surveys will be held on a project folder on a secure server, accessible only to authorized employees. Data will only be shared with project partners in a pseudonymized format, with each cohort participant being allocated a unique identification number. This will be collated in a central database held by IOM for subsequent data analysis. Only members of the research team authorized by the project leader will have access to these databases.

All data related to the project will be retained for at least 10 years for quality assurance purposes.

### Reporting and Participant Feedback

It is not intended to provide participants with details of their individual urinary biomonitoring results as only specific pesticide metabolites will be analyzed, and so we may not assess all pesticide exposure; we can only interpret the results in terms of exposure, not possible ill-health effects. Where a result is unexpectedly high, there will be a review by the scientific advisory board to determine the implications of the result and the need for any action.

The overall study findings will be published as a publicly available report, peer reviewed publications, and conference presentations. The project website will post news about the project at regular intervals, as well as providing access to project publications and conference presentations. Participants will be advised of its URL. Where the original studies include community feedback (Uganda and Malaysia), the summaries of the urine results will be included in these activities.

No personal identifying details of individual participants will be disclosed in any publications or presentations arising from this work.

### Statistical Analysis

#### Evaluation of Recall Bias

Analysis of the collected data will focus on the assessment of participants’ ability to recall information regarding their previous use of pesticides during work over time. Commonly used determinants of exposure to pesticides, as available within study questionnaires, will be compared. These include the duration of use (days and hours per year), methods of mixing and application, products and areas of use, use of PPE during mixing and handling, and personal hygiene activity (eg, timing of personal washing and cleaning clothes). At first, comparisons will involve the newly collected questionnaire data against those already existing within each cohort, stratified by period of recall (ie, short-, medium-, and long-term recall defined as <2 years, 6-12 years, and >15 years since filling out the initial study questionnaire). Subsequently, and whenever possible, data will be pooled together using a standard database management system software (eg, ACCESS). Data pooling will be based on the similarity and meaning of the available questionnaire items across studies, and the included data will be comprehensively reviewed, cleaned, and prepared before the statistical analysis.

Following the initial data cleaning and processing, descriptive statistics will be applied, and the basic attributes of the measurement database will be described. The main analysis will be performed using standard statistical approaches such as the estimation of proportions of agreement and Cohen kappa statistics. The existing data will form the reference category in these comparisons. Standard statistical analysis software (eg, Stata, StataCorp LLC or SAS) will be used.

#### Evaluation of Currently Available Individual-Based Exposure Assessment Methods for Pesticide Exposure

Participants’ daily average exposure will be estimated using the contextual information from the questionnaires and the exposure measurement surveys. Estimations will be based on mathematical equations (algorithms) developed by Dosemeci et al [21] as part of the exposure assessment of the AHS. Improvements were made to these algorithms through updating the assigned exposure-modifying factors and structural components [6,22-24], and a further adaptation of the general version of the AHS algorithm is now available [25].

Exposure predictions will be based both on the original Dosemeci et al [21] and the updated version of the AHS algorithm [25]. However, since the algorithms have been developed specifically for the AHS cohort, tailored adaptations of the updated general algorithm to the exposure situation specific to the populations in question will also be developed, for example, the Uganda cohort already has an adapted algorithm (Fuhrimann, personal communication). As with previous work, tailoring of the algorithms will be based on expert opinion and the available literature.

For each algorithm, 2 sets of intensity scores will be calculated. One will use the information derived from the participant’s questionnaire responses, covering their usual working and exposure practices. The other will use the information collected from the self-reported diaries covering the actual working practices applied during work on the day of the measurements.

The performance of algorithms as tools for assessing exposure to pesticides will be evaluated through comparisons of the estimated intensity scores from all different versions applied on the same sets of information against the results of the collected biomonitoring data. These comparisons are expected to provide information on the exportability of the algorithm exposure assessment approach developed as part of the AHS study to new pesticide exposure populations and situations. The benchmarking of the different versions of the algorithm (ie, original AHS, updated AHS, and population specific) will inform about the gain in performance by the tailoring of the equations to the population and exposure situation at hand.

Data analysis will commence with an assessment of the shape of the distribution of the collected exposure data through graphical means and formal statistical tests. Where sample results are below the limits of detection, proper data processing methods will be selected based on the actual proportion of observed censored values and the available recommendations in the literature [26]. Appropriate transformations will be applied, and exposure measurements and algorithm intensity scores will be summarized using the corresponding central tendency measured. Differences between mean values of continuous variables will be evaluated, depending on the requirements, using paired or unpaired Student *t* tests, or analysis of variance regression for comparisons between multiple groups. If required, nonparametric statistical approaches will be applied. Chi-square tests will evaluate differences between groups in characteristics of a categorical nature. The associations between the different algorithm scores and the exposure measurements will also be explored using conventional regression analysis approaches including correlation analysis. Multivariate linear regression models with the exposure measurements as the dependent variable and the algorithm parameters or estimated scores as the independent variables will also be employed to allow the influence of the different parameters in the exposure to be examined.

Epidemiological studies frequently perform the analysis on the basis of exposure categories derived from the distribution of the objective exposure measurement results (eg, tertiles and quartiles). Therefore, analysis with the exposure intensity scores as a categorical variable will also be performed. Cutoffs for the exposure categories will be based on the distribution characteristics of the derived intensity scores and exposure measurement results. The differences between the means of the measured exposure concentrations between the established categories of scores will be evaluated, and Chi-square tests for independence between the categories of the scores and the measurements will be performed. Classical agreement analysis between categorical variables will also be performed.

#### Power Calculations

Power calculations (see Multimedia Appendix 1) indicate that 150 to 216 questionnaire responses and at least 84 participants providing urine samples are required to provide 80% power for the various comparative analyses. Data from the ongoing PIPAH study suggest an expected response rate of 25% to 40% for the repeated questionnaire. We can therefore reasonably expect 125 to 200 questionnaire responses for the PIPAH study and, given that this is an engaged cohort, we might assume that many may also consent to the exposure assessment project aim. The Malaysian cohort has already recruited and sampled approximately 150 participants. The Uganda cohort will look to recruit >84 participants for the exposure assessment study. For the questionnaire recall, the number of participants recruited from the SHAW and historical biomonitoring populations cannot be guaranteed (owing to age of participants); it may therefore only be possible to analyze these data on a pooled basis.

### Ethical Considerations

As previously stated, each cohort will seek separate ethical approval based on the same outline protocol. The IMPRESS project has also been registered on Research Registry (identification number 4292).

In addition, an advisory board comprising 4 independent experts has been appointed and will monitor the study progress throughout the project. They reviewed earlier versions of the study protocols for the work described in this manuscript.

Studies such as the one summarized in this paper may encounter a number of difficulties with recruitment and with the quality of information recorded by participants. Care must also be taken to avoid research fatigue (participants withdrawing owing to excessive demands from the projects). IMPRESS is based on existing cohorts whose participants have already demonstrated a commitment to participating in research and from that previous or ongoing involvement, they have some idea of what participation will entail. In the United Kingdom, following the initial response to the recall exercise, only 1 reminder will be sent to participants regarding the completion and return of the study material. Where participants expressed an interest in being contacted for the biomonitoring element of the project, after the initial approach, there will only be 2 reminders within 2 months during the spray season. In Uganda and Malaysia, recruitment is only attempted once, at the point of visiting the worksite.

We consider that there are no risks to participants in taking part in the research project, and we will take steps to minimize any burden that they may experience. In particular, the length and language used in the surveys will not be onerous. Participants will be asked to provide urine samples on specific days during the production or growing season which are relevant, where possible, to the use of selected pesticides. The provision of urine samples is not considered difficult or invasive.

The survey materials will not include any topics that might be considered sensitive, embarrassing, or upsetting and criminal or other disclosures requiring action are not considered as possible to occur during the study. If the field researchers observe dangerous practices, then they will advise the individual accordingly (this is not relevant to UK participants as their application practices will not be observed). Owing to the classic observational design (without any intervention) of this study, we also do not anticipate any specific health or other issues arising from it. Hence, there are no specific criteria for suspending or terminating participants in this study.

Finally, participants will not be compensated for their time incurred in participating in the IMPRESS project. This reflects the conditions offered in the original cohort studies.

#### Ethics Approval and Consent to Participate

PUHS and PIPAH: Ethical approval for the study has been obtained from the University of Sheffield’s Research Ethics Committee (REC) for the assessment of recall bias (Reference Number HSL28) and the exposure assessment (Reference Number HSL29). The Greater Manchester Central REC gave approval for the PUHS to share individual-level data collected as part of the 2004-2006 Survey of Pesticide Usage with the PIPAH study (REC Reference number 14/NW/1042).

SHAW: Ethical approval has been obtained from the University of Manchester RECs (2019-5987-9976).

UK Historical biomonitoring data: Ethical approval was granted by the HSE’s Research Ethics Panel (Impress_ERAC_140819).

Uganda: Ethical approval will be sought from Utrecht University in the Netherlands and the Higher Degrees Research and Ethics Committee at Makerere University in Uganda.

Malaysia: Ethical approval for the study has been obtained from the University of Manchester RECs (2017-0439-3979) and a Malaysian Medical REC (NMR-17-424-34635[IIR]).

## Results

The project was funded in September 2017. Enrollment and sample collection was completed for Malaysia in 2019 and is on-going for Uganda and the United Kingdom. Sample and data analysis will proceed in 2020 and the first results are expected to be submitted for publication in 2021.

## Discussion

To our knowledge, IMPRESS is the largest and most comprehensive evaluation of pesticide EAM used in epidemiological studies of working populations ever performed, with previous comparable exercises in farming populations being small either in terms of personal measurements involved (generally below 200 measurements per study) or EAMs or scenarios included [6,22,23,27-31]. The study builds on work already undertaken within the AHS [32], which looked at the impact of misclassification in 83 operatives using 2 active ingredients, concluding that misclassification may result in false-negative findings and hence underestimate exposure risks. It is anticipated that the knowledge obtained from the project will assist in optimizing the way in which epidemiological studies of occupational pesticide exposures perform their exposure assessment. This is probably an important development considering that surrogate EAMs comprise the main exposure assessment in more than 70% of the epidemiological studies published within the last 25 years with increasing trends in use being observed for some of these methods within the same period [8].

## Appendix

Multimedia Appendix 1 Supplementary material – Power calculations.

Multimedia Appendix 2 Peer-reviewer report 1 from IMPRESS.

Multimedia Appendix 3 Peer-reviewer report 2 from IMPRESS.

## Abbreviations

AHS: Agricultural Health Study
EAM: exposure assessment method
GDPR: General Data Protection Regulation
HSE: Health and Safety Executive
IMPRESS: Improving Exposure Assessment Methodologies for Epidemiological Studies on Pesticides
IOM: Institute of Occupational Medicine
NRoSO: National Register of Sprayer Operators
PESTROP: pesticide use in tropical settings
PIPAH: Prospective Investigation of Pesticide Applicators’ Health
PPE: personal protective equipment
PUHS: Pesticide Users Health Study
REC: Research Ethics Committee
SHAW: Study of Health in Agricultural Work
