# Supplementary material for: Improving Exposure Assessment Methodologies for Epidemiological Studies on Pesticides: Study Protocol
Source: JMIR Res Protoc. 2020 Feb 28;9(2):e16448. doi: 10.2196/16448 (PMC7070347; doi:10.2196/16448)
Supplement: Multimedia Appendix 1 [file resprot_v9i2e16448_app1.docx]

***Supplementary material – Power calculations***

***Assessment of recall bias***

The assessment of the recall patterns over time will involve comparisons of paired data collected from the new questionnaires against those already existing within each of the involved cohorts. For binary data (e.g. use or not of PPE during application, mixing or not) the analysis will likely focus on the percentage agreement in responses at the two time points and using the statistical kappa co-efficient (see e.g. [1]). Continuous data would be analysed using t-test for paired values with the null hypothesis being that there is no difference between the estimates collected in the newly and already existing questionnaires.

It is estimated that based on a kappa between 0.3 and 0.6 [1], a study size of maximum 216 participants will be required to achieve a precision of +/- 0.1, (i.e. 80% confidence interval of the form kappa +/- d, where d does not exceed 0.1) assuming that the subdivision or responses between categories (e.g. wearing/not wearing PPE) is no greater 80 % / 20 % [2]. If subdivisions for the same categories were to be in the range of 70 % / 30 % then the same precision will require a sample of 172 persons to be achieved instead (Table 2).

**Table 2.** Numbers of subjects required for the analysis of binary data to estimate kappa’s of different size with a precision of +/- 0.1 (i.e. with an 80% confidence interval).

| **Proportion of responses without a certain characteristic (e.g. not wearing PPE)** | | **Required sample size (n)** | | |
| --- | --- | --- | --- | --- |
| **Original Questionnaire** | **Repeated**  **Questionnaire** | **Kappa = 0.3** | **Kappa = 0.4** | **Kappa = 0.6** |
| 0.1 | 0.1 | 358 | 362 | 302 |
| 0.2 | 0.2 | 216 | 209 | 167 |
| 0.3 | 0.2 | 153 | 168 | 133 |
| 0.3 | 0.1 | 176 | 150 |  |
| 0.3 | 0.3 | 172 | 162 | 126 |
| 0.4 | 0.2 | 135 | 127 |  |
| 0.4 | 0.3 | 153 | 143 | 110 |
| 0.4 | 0.4 | 155 | 143 | 110 |

Paired continuous data would be analysed using t-test for paired values with the null hypothesis being that there is no difference between estimates across the different time points. The sample size required to assess the difference will depend on the variability of the estimates and the size of the difference that the study team want to detect. The more variable the estimates and the smaller the difference, the more subjects will be required. It can be estimated that an average difference in exposure between surveys of three exposure units (EU) with a standard deviation of around 13 EU [3] will require a sample size of 150 subjects would be required to have 80% power to detect a difference of this size at the 5% level of statistical significance. Further sample size estimations required for a range of difference between 1-5 units and standard deviations ranging from 10 to 18 can be seen in Table 3.

**Table 3.** Number of subjects required to detect a certain difference ranging between 1-5 units in a continuous parameter as a function of its measured standard deviation.

|  | **Standard deviation** | | | | |
| --- | --- | --- | --- | --- | --- |
| **Difference** | **10** | **12** | **14** | **16** | **18** |
| 1 | 787 | 1132 | 1541 | 2012 | 2545 |
| 2 | 199 | 285 | 387 | 505 | 638 |
| 3 | 90 | 128 | 173 | 226 | 285 |
| 4 | 52 | 73 | 99 | 128 | 161 |
| 5 | 34 | 48 | 64 | 83 | 104 |

***Evaluation of currently available individual-based EAMs for pesticide exposure***

Exposure estimates and measurement results will be included as continuous variables in evaluations involving correlation and linear regression analysis (for algorithm parameters). Since in epidemiological analysis individuals are frequently grouped in exposure categories based on ordinal scales of intensity (e.g. no, low, medium, high exposure), the algorithm scores and measurements will also be used to create such categories using tertiles or quartiles as cut-offs limits. Comparisons of the mean measured pesticide metabolite concentrations between the established categories using algorithm scores will be performed using ANOVA and two sided t-tests whereas differences between the categories based on the algorithms and those based on the measurements will also be explored using agreement analysis and x^2^ tests.

Previous evaluations of the updated AHS algorithms reported correlations between pesticide metabolite concentrations (2,4-D and chlorpyrifos) and algorithm intensity scores based on questionnaires ranging between approximately 0.30 and 0.50 [4]. Detection of such relationships within our study with a statistical power of 80% will require a maximum of 84 samples (Table 4).

**Table 4.** Number of subjects required to detect a relationship of a magnitude between 0.3 to 0.8 for different probabilities of obtaining a statistical effect (i.e. study power).

| **Study power** | **Effect size** | | | | | |
| --- | --- | --- | --- | --- | --- | --- |
|  | **0.3** | **0.4** | **0.5** | **0.6** | **0.7** | **0.8** |
| 0.50 | 42.5 | 23.5 | 14.5 | 10.5 | 7.5 | 5.5 |
| 0.55 | 47.5 | 26.5 | 16.5 | 11.5 | 8.5 | 5.5 |
| 0.60 | 53.5 | 29.5 | 18.5 | 12.5 | 8.5 | 6.5 |
| 0.65 | 59.5 | 32.5 | 20.5 | 13.5 | 9.5 | 6.5 |
| 0.70 | 66.5 | 36.5 | 22.5 | 14.5 | 10.5 | 7.5 |
| 0.75 | 74.5 | 40.5 | 25.5 | 16.5 | 11.5 | 7.5 |
| 0.80 | 83.5 | 45.5 | 28.5 | 18.5 | 12.5 | 8.5 |
| 0.85 | 95.5 | 52.5 | 31.5 | 20.5 | 14.5 | 9.5 |
| 0.90 | 111.5 | 60.5 | 36.5 | 23.5 | 16.5 | 10.5 |

A comparable and even smaller number of measurements is required for the analysis using the algorithm scores as ordinal variables. In particular, geometric mean concentrations (GSD) for measurements of pesticide metabolite (2,4-D) concentrations across approximate tertiles of the distribution of intensity scores obtained from questionnaires have previously been reported as 13 ug/l (3.5), 19 ug/l (3.0), and 52ug/l (4.3) for the low, medium, and high exposed groups respectively [4]. The agreement between the questionnaire-administered algorithm categories and those from urine metabolite measurements was reported as 50%. Based on these results, a sample size of approximately 40 will be required to allow differences comparable to the smallest (i.e. between low and medium categories) reported by Thomas [4] to be detected with a statistical power of 80% and assuming an increase in variability by a factor 2 in both groups (i.e. GSD of 7 and 6, respectively). Similar sample sizes are also advised in categorical analysis looking to establish significant differences (α=0.05) for effect of 0.5 magnitude with an 80% confidence (Table 5).

**Table 5.** Number of subjects/measurements required to detect a difference in a x^2^ test with effect sizes between 0.1 to 0.6 for different probabilities of obtaining a statistical effect (i.e. study power).

| **Study power** | **n by effect (Cohen’s w) size** | | | | | |
| --- | --- | --- | --- | --- | --- | --- |
|  | **0.1** | **0.2** | **0.3** | **0.4** | **0.5** | **0.6** |
| 0.50 | 642 | 161 | 71 | 40 | 25 | 17 |
| 0.60 | 792 | 198 | 88 | 50 | 32 | 22 |
| 0.70 | 968 | 242 | 107 | 61 | 39 | 29 |
| 0.80 | 1193 | 298 | 133 | 75 | 48 | 33 |
| 0.90 | 1541 | 385 | 171 | 96 | 62 | 43 |

**References**

1. Blair, A., et al., *Reliability of reporting on life-style and agricultural factors by a sample of participants in the Agricultural Health Study from Iowa.* Epidemiology, 2002. **13**(1): p. 94-9.

2. Cantor, A.B., *Sample-Size Calculations for Cohen's Kappa*, in *Psychological Methods*. 1996. p. 150-153.

3. Negatu, B., et al., *Occupational pesticide exposure and respiratory health: a large-scale cross-sectional study in three commercial farming systems in Ethiopia.* Thorax, 2017. **72**(6): p. 498-499.

4. Thomas, K.W., et al., *Assessment of a pesticide exposure intensity algorithm in the agricultural health study.* J Expo Sci Environ Epidemiol, 2010. **20**(6): p. 559-69.
