# Supplementary material for: Improving Exposure Assessment Methodologies for Epidemiological Studies on Pesticides: Study Protocol
Source: JMIR Res Protoc. 2020 Feb 28;9(2):e16448. doi: 10.2196/16448 (PMC7070347; doi:10.2196/16448)
Supplement: Multimedia Appendix 2 [file resprot_v9i2e16448_app2.pdf]

IMPRESS: Improving exposure assessment methodologies for epidemiological studies on pesticides

Date: 2nd August 2019

Manuscript Title: IMPRESS: Improving exposure assessment methodologies for epidemiological studies on pesticides

ECPA comments on draft above manuscript received by Karen Galea: 7th June 2019

The IMPRESS project team provide their responses to the main ECPA comments below. ECPA comments that were made within the draft manuscript itself are addressed in the track-changed version of the final manuscript.

Main comments:

Comment 1: WP2 recall of past PPP exposure and determinants – the proposal details this will utilise the PIPAH study, The Thai Farmers' families study, The Ethiopian Farm Workers Study plus the PUHS. In the manuscript there is no mention of the Thai study but instead, it seems that there is some discussion of an Ugandan study plus the completed Malaysian study. Could you clarify?
Response: Indeed according to our initial proposal a study among Thai farmers previously completed by UoM was included as part of the WP2 work. Very late 2017 and shortly before our first AB meeting it became evident that re-establishing the specific cohort was going to be too difficult and logistical costly and thereby it was decided to omit this study from WP2. Instead, we decided to use the data collected from Thai farmers study to tailor the algorithms currently being developed for the Malaysia study. The Ugandan study, as communicated also on our last in person meeting, was proposed as an alternative to the Ethiopian study amid our difficulties to (re-)establish the specific cohort. At present it is evident that we will be able to perform the WP3 activities in Ethiopia, not WP2 (which is the follow-up of farmers who participated in the previous study). The Uganda study will contribute to both WP2 and 3 of the IMPRESS project.

Comment 2: Then, the original proposal states "*re-interviewing participants from the above studies will allow us to test the reliability and recall over time (Task 2.3). During interviews focus will be given on the exposure determinants identified through the review process in WP1*". This goes back to the comments we made on the manuscript for WP1 which ended up being a literature review rather than a critical review of and identifying the best EAMs/exposure determinants. So a question is what are the identified exposure determinants that will be focused on in WP2?
Response: The original proposal description envisaged the re-interviewing of participants on specific items of the questionnaires (e.g. type of mask used during spraying; or spraying equipment). Following in-depth discussions within the Management Committee (MC) and liaison with the Advisory Board (AB), it was decided that we employ the questionnaire methodology and approaches as close as possible to the original administrated ones. This included the actual content of the questionnaires in terms of questions and structure as to avoid changes in context that could potentially bias study results. Effectively, across all included studies, the complete section of the questionnaire relevant for current pesticide exposures as administered at baseline has been included. Non-essential sections for the purposes of the IMPRESS project (e.g. questions on health

symptoms, risk perception, demographics or employment history) were removed. The unrestrictive repeated use of all relevant questions within a study makes the determinant identification process *a priori* irrelevant. The data analysis can still focus on certain determinants. If this is required (e.g. for logistical reasons)we would select and include those factors comprising parameters of the algorithm given their selection for inclusion on the algorithm is based on evidence on their importance sourcing from the literature.

Concerning the composition of the WP1 paper, we would like to point to our previous personal and in-writing communications regarding the specific concern. In brief, and as previously explained, our review work related to the applied methods has been effectively divided into two separate papers: a) the WP1 review on used methods in epidemiological study and b) a review paper on the information available regarding the validity of currently available exposure assessment methods in occupational epidemiology (an outcome of the previously funded ECPA project). The two papers are currently considered for publication in peer-reviewed literature and cross-referenced between them.

Comment 3: Additionally in WP2 the project team stated they *will identify data sources, records within existing studies or other sources of information (Task 2.1)*. The only additional information sources we can see in the draft manuscript are from the U.K.? If this is indeed the case does the scope of additional work need to be widened given the scope of the IMPRESS project?
Response: On the contrary, besides the additional UK data we have already included a new African study – i.e. the Ugandan cohort. We have also explored the possibility of using existing AHS data with little success amid that the relevant data have already been thoroughly described and published in the literature. Reasons for the exclusion of the Thai and Ethiopia studies from WP2 have already been provided.

Comment 4: WP3 assessing the reliability and validity of individual-based EAMs. The original proposal states "*The aim of this WP is to assess the reliability and validity of the most advanced individual-based exposure assessment methods available to date comprising of a semi-quantitative approach based {an} algorithm utilising existing information on exposure determinants such as mixing conditions, duration and frequency of application, application methods, maintenance or repair of mixing and application equipment and work practices*". This sentence is confusing as exposure assessment methods (i.e. plural methods) are mentioned but then only the AHS based algorithm is discussed. This is reflected in the draft manuscript where only the AHS and an amended AHS algorithm is discussed. Because WP1 didn't actually identify the 'best' EAMs we do think that the project team currently lacks the rationale of using the AHS algorithm. However, if it is assumed that the AHS algorithm is the 'best' EAM then fair enough – otherwise this position needs to be better defended.
Response: The "AHS" is a series of different algorithms developed under the same principle but with a completely different composition in relation to exposure affecting factors and related size effects (weighing factors) as well as in relation to the time frame concerned (i.e. exposure in a specific day, average or regular). The inference as "AHS algorithm" of all different mathematic equations available is made for simplicity reasons as the one developed for the AHS study in early 2000 is the most famous one given also the scientific impact of the study within which it has been developed. The choice of the algorithms for inclusion in WP3 evaluations sources from the fact that the algorithm is the most comprehensive attempt to develop a method for individual exposure assessment of pesticides documented also from the several validation exercises performed on previously. In addition, the validation exercises performed as part of WP3 make the specific algorithm a good reference for the benchmarking exercises planned to be performed as part of WP4 that will include other group based exposure assessment methods. We need note that in presence of a large day-to-day variability in exposure, as with pesticides, literature suggest that estimation of

long term exposure on the basis of individual exposure assessment may be preferred[1]. The importance of the AHS algorithm as an exposure assessment method for epidemiological studies in pesticides has been stressed before both in opinion[2] and review[3] papers. At present we do not feel that we need to further elaborate on the above within the protocol paper particularly considering that methods will be more detailed described within the papers summarising the actual results of the study. Nevertheless we do realised the importance of highlighting the fact that WP4 will not be limited on evaluating performance of the AHS and thereby we have altered the relevant text in objective 2 of the "Protocol aims and objectives" section.

Comment 5: Finally: **Timelines**. From the revised project timelines, our understanding is that the extended project completion date is August 2021. In the draft manuscript the recruitment for the Ugandan and Ethiopian studies is stated as being 2019/20. If recruitment and sampling takes place in early to mid-2020 then this only gives a very short time period to get all the samples shipped, analysed and reported so overall statistics and project conclusions etc and final output can be done on time. Would you be able to provide your view for future timelines of this project?

Response: According to our research planning and projected timelines the work in Uganda and Ethiopia is projected to commence with enrolment of farms between August and September 2019. Field work in Uganda is to commence in mid-October and in Ethiopia by November 2019. It is expected to be finalised by January 2020 which still remains within our planned and previously communicated timescales both to the AB and funder. We also need note that Milestone achievement at present is on track with the DTAs currently being reviewed prior final approval and implementation whereas progress in other studies and WP1 and WP4 are also on track.

[1] Preller et al 1995. Modeling long-term average exposure in occupational exposure-response analysis. Scand J Work Env. Heal. 21, 504–512

[2] Kromhout et al 2005. Effects of errors in the measurement of agricultural exposures. Scand J Work Env. Heal. 31 Suppl 1, 33–37

[3] Basinas et al., 2019. What is the validity of surrogate measures of occupational pesticide exposure used in epidemiological studies? A review of the published literature. Annals of Work Exposure and Health, submitted
