# Supplementary material for: Improving Exposure Assessment Methodologies for Epidemiological Studies on Pesticides: Study Protocol
Source: JMIR Res Protoc. 2020 Feb 28;9(2):e16448. doi: 10.2196/16448 (PMC7070347; doi:10.2196/16448)
Supplement: Multimedia Appendix 3 [file resprot_v9i2e16448_app3.pdf]

IMPRESS: Improving exposure assessment methodologies for epidemiological studies on pesticides

Date: 24th May 2019

Manuscript Title: IMPRESS: Improving exposure assessment methodologies for epidemiological studies on pesticides

The IMPRESS project Advisory Board provided comments on the draft above manuscript received by Karen Galea, 12th April 2019.

The IMPRESS project team provide their responses to the main comments below (highlighted in red).

Comments on "IMPRESS: Improving Exposure Assessment Methodologies for Epidemiological Studies on Pesticides" from Scientist Emeritus Aaron Blair, supported by Emeritus Professor Len Levy and Dr. Mark Montfort (IMPRESS project Advisory Board members)

General Comments:

This manuscript provides an excellent overview of a project that is likely to provide considerable new and important information regarding the reliability and validity of exposure assessment techniques for epidemiologic studies of pesticides and the variation that might occur in different exposure situations. A very useful outcome of this study will be in the interpretation of existing studies and in the design of future investigations.

Thank you

Some additional discussion about the comparison of the exposure estimates with the measurement data would be helpful. Both could be very accurate, but relatively uncorrelated. This could occur because the two indicators may represent very different time frames, i.e., the monitoring data, which reflects only a few days of very recent pesticide exposure, while the estimates typically reflect a much longer time period. This could be evaluated by specifically asking the participants about the time period that is relevant only for the monitoring data. This is probably planned, but I did not see is specifically spelled out in the manuscript.

The study involves collecting exposure information related to the day of sample collection. Two sets of intensity scores will be calculated; one on "usual practice" and one on the actual information provided for that day (see p15). This will provide information on how useful a day's exposure assessment can be for long-term exposure assessment.

Research Team – This research team is very experienced regarding exposure assessment in general, as well as specifically for pesticides.

Advisory Board – We meet annually with the research team to provide advice regarding study plans. Members of the Advisory Board reviewed this manuscript and provided individual comments. We are supportive of this project.
Amended

The manuscript will be of interest to the exposure assessment community.

Specific Comments:
Page 2 – You might consider modifying the phrase "large number of pesticide active ingredients". You see this a lot in studies of populations with pesticide exposures and I think it conveys the opinion that pesticides are different from most occupational exposures. I actually think the number of pesticide exposures possible for "individuals" in any single pesticide-using situation is typically smaller than the number of chemical exposures possible for many factory workers. There are many, many pesticides, but most people only are only dealing with a few commodities/pests, so they only have contact with a limited number of the pesticides on the market. In the industrial setting I have typically found workers are usually exposed to hundreds of different chemicals.
Amended.

Page 2 – Should mention that use of protective equipment and personal hygiene is sometimes included in exposure assessment algorithms.
Amended.

Page 3 – Pesticides have been associated with effects on the respiratory system. Added with reference.

- Might modify the sentence regarding availability of measurement data to "measurement data are rarely available to adequately cover the entire exposure time period" Amended
- Point 4 is not clear to me. We do acknowledge that the terms "individual-based" and "group-based" exposure assessment may be confusing for readers. We have now rephrased the sentence to improve clarity including definitions to the above.

Page 4
– Point 1 – Might want to name the populations studied, or refer to "those studied here"
Amended

Page 14 – Exposure prediction will be based on the first and updated algorithm from the AHS and an AHS version modified by IMPRESS investigators to tailor it to the populations being studied by IMPRESS. Does the "benchmarking of the different versions" refer to three versions, i.e., two directly from the AHS and one AHS as modified for a specific population in this study?
Yes, a clarifying clause has now been included in the relevant sentence.

Page 15 – Need to give some indication of the size of the differences that can be detected with 80% power.

Power calculations involve estimates for range of potential outcomes across different types of analysis. This is explored in detail in the Supplementary Material and as such we feel that is unnecessary to include a level of such detail on the manuscript itself. After all this was the main reason that we decided to remove the relevant part from the main text.

Page 16 – Might cite the other exposure evaluations that have been performed here and to indicate their relative size to give the reader a clear indication of the increase in size of this effort.
A short statement referencing previous comparable exercises have now been included.

-Might also refer to the paper "Blair A, Thomas KT, Coble J, Sandler DP, Hines CJ, Lynch CF, Knott C, Purdue MP, Zahm SH, Alavanja MCR, Dosemeci M, Kamel F, Hoppin JA, Beane Freeman L, Lubin JH. Impact of pesticide exposure misclassification on estimates of relative risks in the Agricultural Health Study. Occup Environ Med 2011;68:537-541" which deals with exposure misclassification and its effects on relative risks for the AHS, since the AHS algorithm is an important component of this methodologic work. Added.
Page 17 – My title should be "Scientist Emeritus" not "Emeritus Professor". You could use my degree designation "Dr" as you did for Mark. Amended

Comments on "IMPRESS: Improving Exposure Assessment Methodologies for Epidemiological Studies on Pesticides" from Professor Silvia Fustinoni (IMPRESS project Advisory Board member)

A general positive comment is deserved to the manuscript that states the aim of the IMPRESS project and details the methodology.
Thank you.

I agree with comments made by Aaron and supported by Len and Mark. Find here some addition comments/suggestions from my side:

Abstract- Background. I suggest changing the sentence starting with IMPRESS will also evaluate………and the associated health effect. I do not see that this project will perform work on health effects. Maybe the sentence can be rephrase as:
"IMPRESS will also evaluate the size ~~and effect~~ of recall bias on the misclassification of exposure to pesticides; this in turn will affect epidemiological estimates of the effect of pesticides on human health"
Amended

Abstract-methods/design.
In the sentence "the reliability of pesticides exposure recall over different time-periods will be evaluated" you can add a range of years for the different time periods.
Added

Introduction

Page 3. The sentence "Pesticide exposure intensity has also been understudied/under accounted for but……" is interesting, but I do not see how the work can deal with this issue.
Intensity scores are to be calculated (p15). We appreciate that our statement has been misleading. Our intention was to highlight that this complexity in assessing exposure is likely one of the main reasons for the lack of an adequate quantification of the exposure response relationships. We have now clarified this.

Page 3. The reference Ohlander et al. is missing from the reference list.
Added.

The two aims: I suggest being consistent in the way the 2 aims are called in the manuscript. In particular, while the first aim is consistently called "recall", there are different ways of calling the second aim: I suggest choosing "exposure assessment", as in Table 1. Moreover, I suggest changing the use of "both" in Table 1, indicating the two aims. In the present form, Table 1 is not self-explaining.
Amended

Last sentence of Page 5-beginning of page 6. I think a reference to support the evidence described is necessary.
Reference added.

Methods
Page 7. Here you can see that the second project aim is called "reliability and validity" instead of exposure assessment.
The same hold true in page 8, UK cohorts, third paragraph
All second project aim mentions updated.

Page 9. Data collection. Here you mention a Cambridge Cognition Examination Instrument. You should say way you want to use it in the SHAW farmers; and what about the subjects of the historical biological monitoring groups? I bet they are quite old as well. Maybe you should set an age cut off for the use of this instrument.
The applied IMPRESS sub-protocol follows closely the one applied for each of the involved studies including the format and context of the questions asked. The Cambridge cognition examination instrument was part of the original study protocol amid to assess cognition for this workers given their advanced age during enrollment. At present it is expected that all SHAW participants will be >60 years of age and well beyond retirement and which further validates the use of the specific tool within SHAW. For the remaining cohorts, and including the historical biological monitoring studies, the specific tool is of lesser importance given that the target participants are those still active professionally.

Page 9. Evaluation of currently available individual-based EAMs for pesticide exposure.
I suggest to avoid the use of EAM in the title.
Amended.

Page 10. First line. Add a brief explanation for the collection of field blanks.
Added.

Page 11. Table should be Table 2. The legend you should mention biomonitoring and exposure assessment.
Amended

A valuable information in this table would be the half-life of the chosen biomarkers.
We feel that this is unnecessary. All are modern active ingredients with elimination half-lives of <24 hours. It would, additionally, over-complicate the table.
